# Determination of ^2^H KIEs from Competition Experiments: Increased Accuracy via Isotopic Enrichment

**DOI:** 10.1007/s11244-017-0740-1

**Published:** 2017-05-10

**Authors:** Chiara Colletto, Daniel Whitaker, Igor Larrosa

**Affiliations:** School of Chemistry, University of Manchester, Oxford Road, Manchester, M13 9PL UK

**Keywords:** Kinetic isotope effect, Competition experiments at natural abundance and/or in isotopically enriched substrates, Quantitative ^2^H NMR analysis

## Abstract

Methods for the determination of ^2^H KIEs at natural abundance of deuterium have not been widely used due to the requirement for very long NMR times or large reaction scale. We previously reported a simple methodology for reducing these restrictions by the addition of a small amount of deuterated substrate. Herein, we evaluate the deuterium loadings that give the lowest errors in the determination of ^2^H KIEs. Our simulations indicate that our approach leads to a 4000-fold reduction in NMR time over natural abundance methods.

## Introduction

**Fig. 1:**
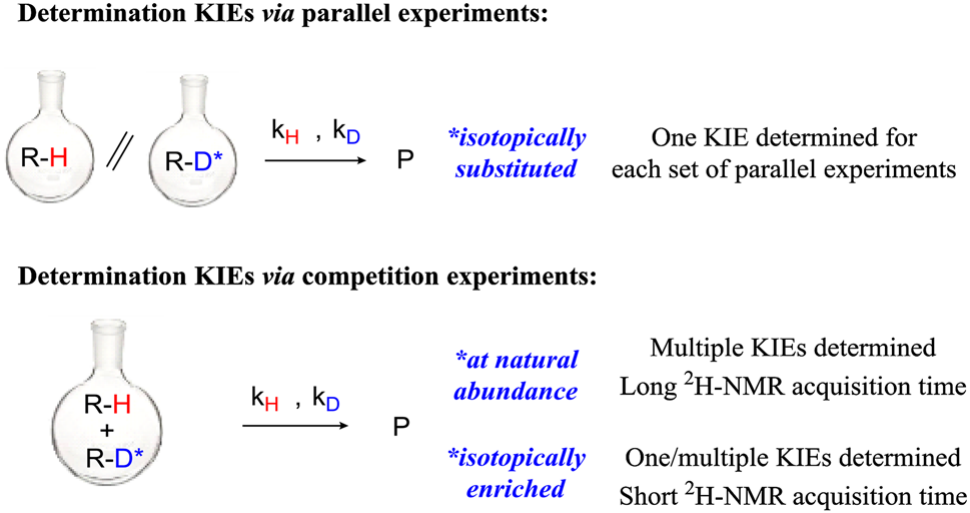
Typical experiments for the determination of ^2^H -KIEs

Determination of the ^13^C and ^2^H kinetic isotope effect (KIE) is a powerful and widely applied tool for gaining insights into the mechanism of organic and organometallic reactions [1]. In particular, KIEs can give useful information regarding which bonds are being rehybridized, broken or formed during the rate determining step of the reaction [2, 3]. Traditionally, KIEs are either obtained through parallel reactions or intermolecular competitions of isotopically substituted substrates [4]. In the former case, KIEs are determined as the ratio of the reaction rate constants (*k*_H_/*k*_D_) determined by kinetic analysis. In the case of intermolecular competition experiments, the KIEs are related to the ratio of the products obtained from the two isotopically different starting materials (i.e. P_H_/P_D_), normally using two different substrates with equivalent reactivity so that this ratio can be evaluated. In both cases, the set of experiments can only lead to the determination of the KIE of the X–H bond that has been isotopically substituted. Singleton and co-workers have demonstrated that the KIE values of multiple H (or C) can be determined simultaneously using a competition experiment at natural abundance of the heavy isotope (Fig. 1) [5, 6]^1^. During the course of the reaction, positions in the starting material subject to a KIE will exhibit a change in the amount of heavy isotope. In this way, unreacted starting material will progressively become heavy atom enriched at positions with a positive KIE and depleted at positions with an inverse KIE. Quantitative NMR acquisitions of ^2^H (or ^13^C) nuclei of the reactive substrate before and after the reaction will lead to different relative integrations. This difference (R/R_0_) is related to the conversion of the reaction (F) and the KIEs through Eq. 1 [5, 6].

1 $${\text{KIE }}={\text{ }}\frac{{{\text{ln(1-F)}}}}{{{\text{ln[(1-F)R/}}{{\text{R}}_{\text{0}}}{\text{]}}}}$$

In recent years, determination of ^13^C KIEs via competition experiments at natural abundance has become a routine method for probing the structures of transition states and intermediates^2^. On the other hand, simultaneous determination of ^2^H KIEs at natural abundance has been poorly applied due to the requirement of an extensive acquisition time for quantitative ^2^H NMRs (the natural abundance of ^2^H is 0.0156% compared to 1.107% for ^13^C)^3^. The first simultaneous determination of ^2^H KIEs was reported by Singleton et al. in 1995 [6], where the transition state of the Diels–Alder reaction between isoprene and maleic acid was investigated. In order to acquire the quantitative ^2^H NMR spectra, it was necessary to perform the reaction using ~13 mmol of isoprene, recovering ~4.5 mmol of isoprene for the NMR analysis. The requirement for a large scale reaction becomes problematic when valuable starting materials are used. Recently, our group reported the determination of ^2^H KIEs using isotopically enriched substrates [19]. This allowed the accurate determination of KIEs using NMR without the requirement of an excessive scale up of the reaction or lengthy NMR acquisitions. The aim of the current article is to provide a primer for the use of this method as a simple alternative to natural abundance methods, as well as identifying a reasonable level for deuterium incorporation.

## Determination of KIEs Using Isotopically Enriched Substrates: An Example

This methodology for KIE determination allows studying a reaction under the standard reaction conditions, with the minor adjustment that small amounts of substrates which have been monodeuterated at the positions of interest are added. A reasonable amount of each deuterated material to add is 1–5 mol % (*vide infra*). Any number of substrates deuterated in different positions can be added, allowing the simultaneous determination of several KIEs from one experiment. The KIE is calculated using the method reported by Singleton et al. [6] – the reaction is run until ~65–70% conversion (F, determined exactly by aliquots from the reaction mixture), the starting material is purified from the crude reaction mixture and analyzed by quantitative ^2^H NMR, with the concentration of the deuterated material evaluated by comparison with an internal standard. This concentration (and therefore proportion of deuterated to non-deuterated substrate) is compared with the initial concentration of deuterated substrate. The KIE is evaluated using Eq. 1.

**Scheme 1:**
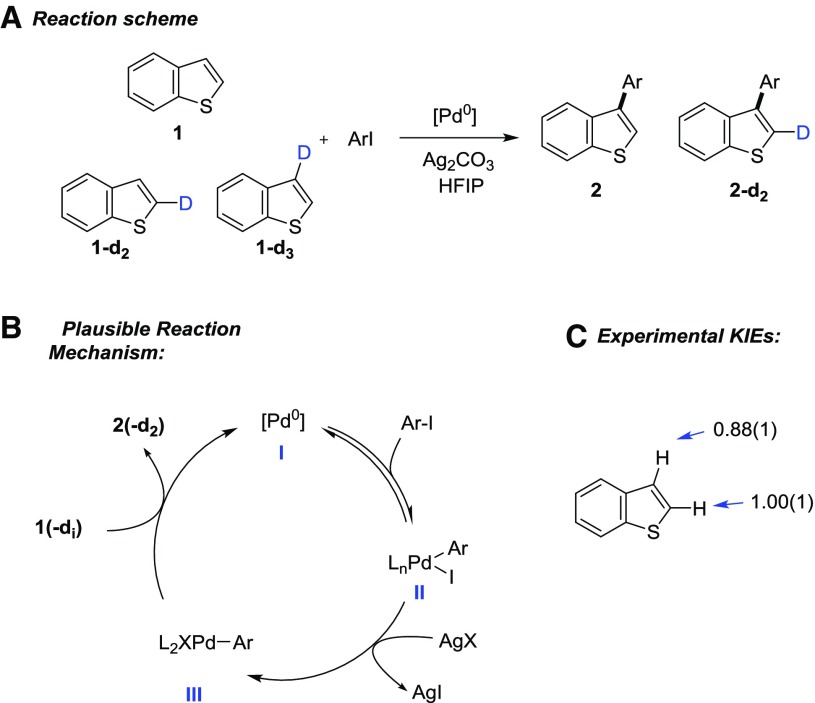
**a** Reaction scheme, **b** plausible catalytic cycle, **c** values of ^2^H-KIEs for the C-2 and C-3 protons of benzo[*b*]thiophene

As an example, in our previously reported β-arylation of benzo[*b*]thiophene the reaction mixture was doped with 1 mol % of substrates **1**-**d**_**2**_ and **1**-**d**_**3**_ (Scheme 1). The reaction was run until 68.2 ± 0.6% conversion, and the KIEs were found to be 0.88 ± 0.01 (C-3 proton) and 1.00 ± 0.01 (C-2 proton). The use of isotopically enriched substrates enabled the acquisition of quantitative ^2^H NMR in relatively short times using only ~0.45 mmol of benzo[*b*]thiophene.

## Evaluating the Error in the KIE

As shown in Eq. 1, KIEs are related to F, the conversion, and R/R_0_, a measure of the change in the ratio of isotopically substituted substrate to unsubstituted substrate (Eqs. 2–3).

2 $${\text{F}}=1 - \frac{{{\text{[1]~}}}}{{{{\left[ 1 \right]}_0}}}$$

3 $$\frac{{\text{R}}}{{{{\text{R}}_0}}}=\frac{{[1{\text{-}}{d_i}{\text{]~~}}{{\left[ 1 \right]}_0}}}{{{{[1{\text{-}}{d_i}{\text{]}}}_0}\left[ 1 \right]}}$$

In order to obtain reliable values of the KIE, the error ΔKIE must be low. ΔKIE derives from a combination of the errors related to the measurement of the conversion (ΔF) and of the concentration of the isotopically substituted substrates (ΔR/R_0_), as reported by Melander and Saunders (Eqs. 4–6) [5].

4 $$\Delta \text {KIE}_\text{F} = \Delta {\text F}~\frac{{\partial\text{KIE}}}{{\partial {\text F}}} = \frac{{ - \ln \left( {{\text R/\text{R}_{0}} } \right)~~\Delta {\text F}}}{{\left( {1 - {\text F}} \right)\ln ^{2} \left[ {\left( {1 - {\text F}} \right) {\text R/\text{R}_{0}} } \right]}}$$

5 $$\Delta \text {KIE}_\text{R} = \Delta \left( {{\text R/\text{R}_{0}} } \right)\frac{{\partial \text {KIE}~~}}{{\partial \left( {\text{R/R}_{0 } } \right)}} = \frac{{ - \ln \left( {1 - {\text F}} \right)~\Delta {\text R/\text{R}}_{0} }}{{\left( { {\text R/\text{R}}_{0} } \right)\ln ^{2} \left[ {\left( {1 - {\text F}} \right) {\text R/\text{R}_{0}} } \right]~}}$$

6 $$\Delta {\text {KIE}} = \sqrt {\Delta {\text {KIE}_{{\text F}} ^{2}} + \Delta {\text {KIE}_{{\text R}} ^{2}} }$$

To evaluate ΔF a “reasonable possible error” of 5% of the conversion has been used to account for several sources of error in the measurement of F that are difficult to quantify, including: imprecision in the NMR integrations due to the vagaries of the spectrometer; comparing inequivalent multiplets; inaccuracy in the measurement of F due to the presence of impurities that could affect the integrations; experimental error arising from weighing both substrate and internal standard; signal to noise error in the NMR instrument. The values of ΔR/R_0_ have instead been estimated based on extrapolations from our empirical data^4^. The error in R/R_0_, ΔR/R_0_, is related to the relative error in the ^2^H NMR integrals for **1**-**d** (Eq. 7). This relative error is equal to the absolute error in the integral, Δ*I*, divided by the magnitude of the integral, *I*. The absolute error in an NMR integral, Δ*I*, is related to the spectral noise, the number of points in the integral and the total number of points in the spectrum [20]. Comparing different spectra of the same compound, using the same acquisition parameters (and assuming there are no changes in shim or temperature), should give predictable changes in Δ*I*. The number of points in both the integral and spectra should be constant when comparing different spectra of the same compound, provided the same integral width is considered. The relative error in the integral is inversely proportional to the concentration of deuterated substrate in the NMR sample (Eq. 8), and proportional to the square root of the number of scans run. Since the total mass of substrate weighed out for the NMR sample is constant, the total concentration of substrates in the sample is approximately constant (*c*, Eq. 9), the concentration of **1**-**d**_**i**_ in the NMR sample is expressed by Eq. 9. Putting this into Eq. 8 and introducing a scaled constant of proportionality *a* gives Eq. 10. We have used our experimental errors to calculate the value of *a* 5, then applied Eq. 7 to evaluate ΔR/R_0_.

7 $${{\Delta }}{\text{R/}}{{\text{R}}_{\text{0}}}={\text{R/}}{{\text{R}}_{\text{0}}}\sqrt {{{\left( {\frac{{{{\Delta }}{{{I}}_{1{\text{-}}{d_i}}}}}{{{{{I}}_{1{\text{-}}{d_i}}}}}} \right)}^2}+{{\left( {\frac{{{{\Delta }}{{{I}}_{{{\left( {1{\text{-}}{d_i}} \right)}_0}}}}}{{{{{I}}_{{{\left( {1{\text{-}}{d_i}} \right)}_0}}}}}} \right)}^2}}$$

8 $$\frac{{{{\Delta }}{{{I}}_{1{\text{-}}{d_i}}}}}{{{{{I}}_{1{\text{-}}{d_i}}}}}=\frac{{{{\Delta }}[1{\text{-}}{d_i}]}}{{[1{\text{-}}{d_i}]}}~ \propto ~\frac{1}{{{{[1{\text{-}}{d_i}]}_{NMR~sample}}~}}~$$

9 $${[1{\text{-}}{d_i}]_{NMR~sample}}=~\frac{{[1{\text{-}}{d_i}]}}{{\left[ 1 \right]}}~ \times {{c}}~$$

10 $$\frac{{{{\Delta }}{{{I}}_{1{\text{-}}{d_i}}}}}{{{{{I}}_{1{\text{-}}{d_i}}}}}~ \propto ~\frac{{\left[ 1 \right]}}{{[1{\text{-}}{d_i}] \times {\text{c}}}}=~\frac{{{{a}}\left[ 1 \right]}}{{[1{\text{-}}{d_i}]}}$$

## Results and Discussion

### A Model for the β-Arylation of Benzo[*b*]thiophene

The proposed reaction mechanism for the arylation of benzo[*b*]thiophene is reported in Scheme 1b. Catalyst **I** undergoes a reversible oxidative addition with an aryl iodide leading to species **II** followed by halide abstraction with a silver salt. C–H functionalization steps involving benzo[*b*]thiophene **1** (or its deuterated analogues **1**-**d**_**2**_ or **1**-**d**_**3**_) and species **III** close the cycle to regenerate I. This catalytic cycle has been considered for the kinetic simulation^6^.

### Simulation of ^2^H-KIE Errors in the β-Arylation of Benzo[*b*]thiophene

**Fig. 2:**
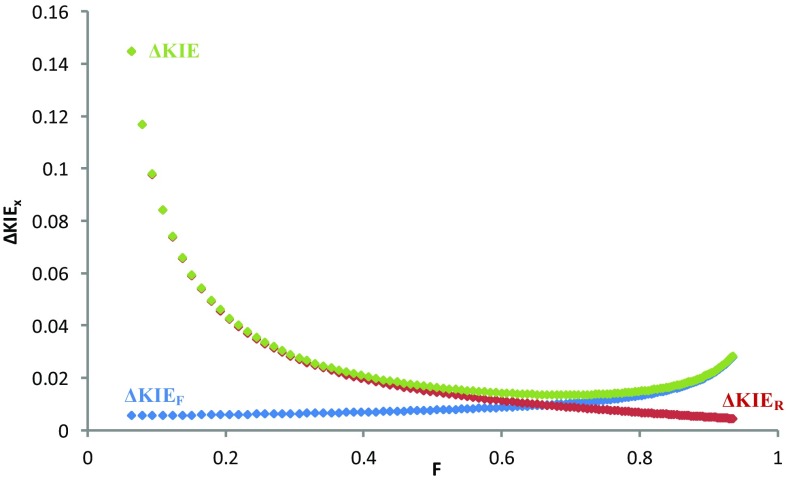
Simulation of errors in KIE measurement for the system decribed in Scheme 1 at different conversions

The calculations related to the determination of the KIE errors have been set to a conversion of 68.2 ± 0.6%, the same as our experimental value. *In silico*, we have demonstrated that the lowest error in the KIE can be obtained at values of F between 60 and 80%. At lower conversion, the greater contribution is associated to ΔKIE_R_, while at higher values of conversion ΔKIE_F_ becomes prominent (Fig. 2).

**Fig. 3:**
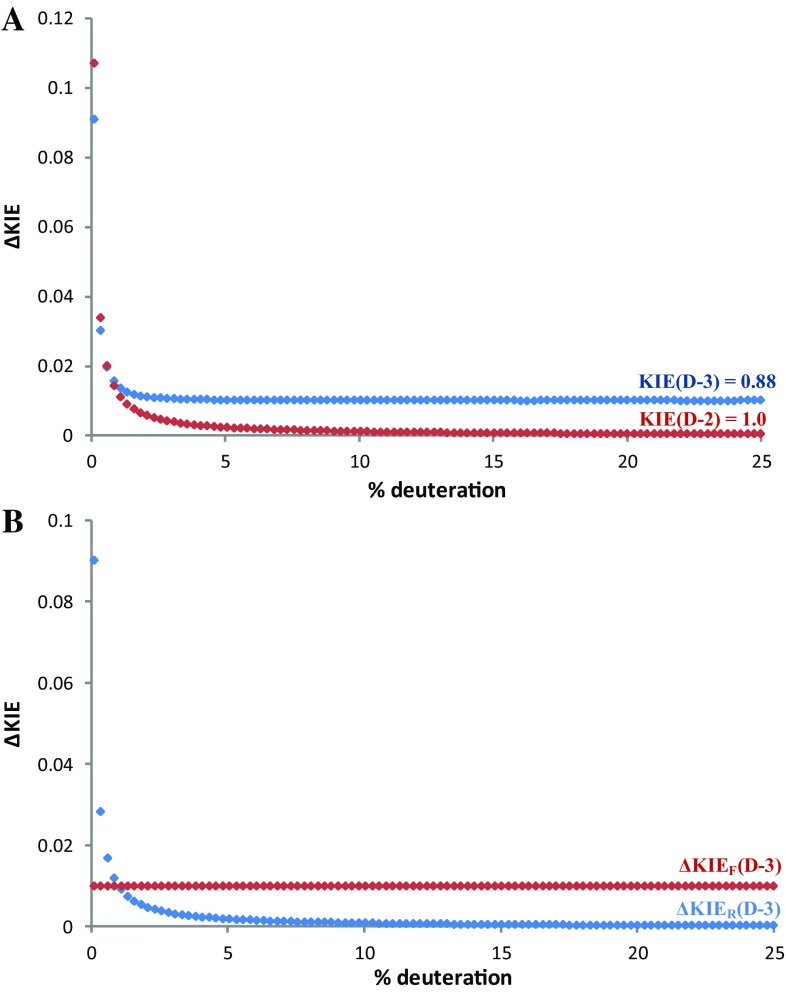
Simulation of errors in KIE measurement for the system decribed in Scheme 1, at 68% conversion. ΔKIE on measured KIE at C-2 and C-3 vs the amount of added deuterated material (**a**). The contributions to ΔKIE (ΔKIE_F_ and ΔKIE_R_) at different levels of deuteration when KIE = 0.88 (**b**)

We then investigated the effect of changing the amount of **1**-**d**_**1**_ and **1**-**d**_**2**_ present at the beginning of the reaction [20]. As the concentration is lowered to the natural abundance level the error in the measurement increases very rapidly due to increases in the NMR error in evaluating the concentration of the deuterated species. This behavior was evident for both KIE values here (0.88 and 1 for C-3 and C-2 position of benzo[*b*]thiophene respectively; Fig. 3a).

On the other hand, increasing the initial concentration of deuterated substrate leads to a decrease in the error, greater for a value of 1 than of 0.88. This is associated to the contribution of ΔKIE_F_, which is the principal source of error at higher concentration of deuterated substrate (Fig. 3b). A KIE of 1 leads to a constant ratio of isotopically substituted substrates, so R/R_0_ is 1 and ΔKIE_F_ = 0 (Eq. 4). These data suggest that low errors in the KIE can be obtained with a minimal loading of deuterated substrate of 1–2%.

### Simulation of ^2^H-KIE Errors in a Generic 2 Substrate Model

**Scheme 2:**
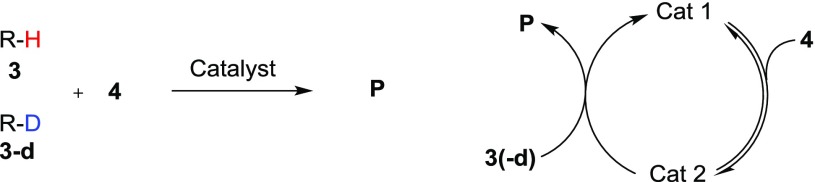
2-Substrate model catalytic system

Interested by these results, we expanded our investigation into other KIE values to see if the same trends would be observed. We chose to use a slightly simplified system for this model, looking at one KIE at a time for a 2-substrate catalytic system (Scheme 2).

**Fig. 4:**
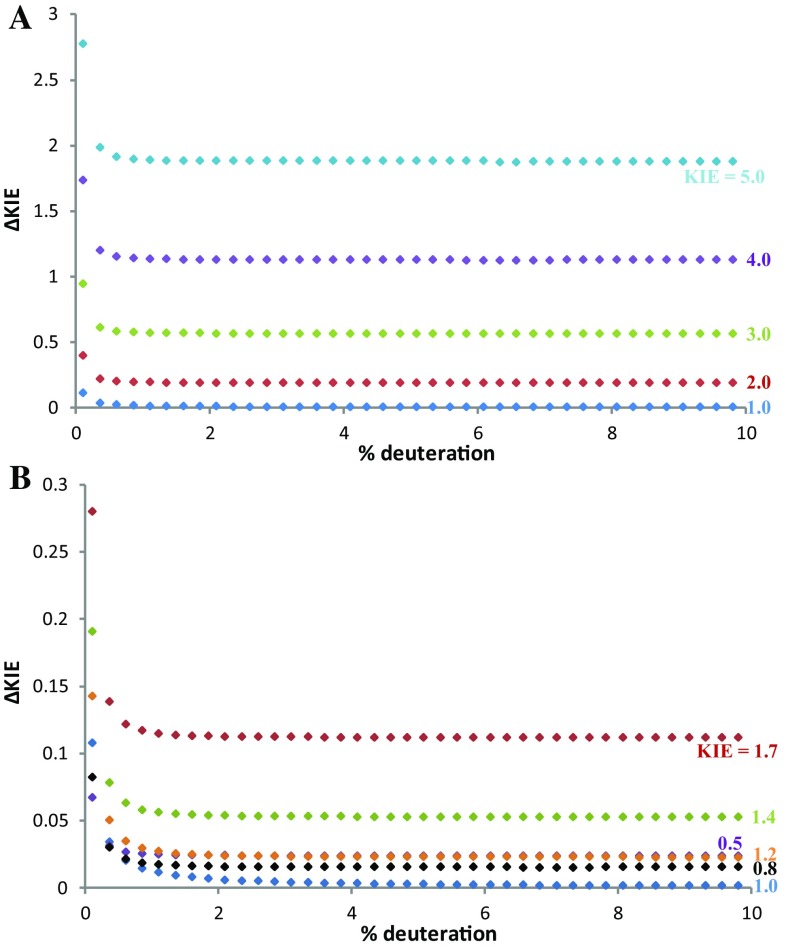
Variation of ΔKIE with different amounts of deuterated substrate for primary KIEs (**a**) and secondary and inverse KIEs (**b**)

The same behavior described above is visible for primary, secondary and inverse KIEs, i.e. the errors reach a plateau around 1–2% of deuterated substrate. The magnitude of the error follows the same trend that has been previously reported, with large kinetic isotope effects having much larger errors (Fig. 4) [5]. The magnitude of the error observed with larger deuterium loading is related almost exclusively to the error in the conversion.

The use of more than ~2% deuterium has no additional effect on lowering the error in the KIE. Very high deuterium loadings would lead to large inaccuracies in the determination of F (due to the low concentration of non-deuterated starting material), but use of 2% of each deuterated substrate would allow several deuterium KIEs to be simultaneously evaluated without loss in accuracy.

Finally, we used our model to simulate the amount of NMR time (expressed as the number of scans) that would be required to obtain comparable errors for KIEs evaluated using the natural abundance of deuterium.

**Fig. 5:**
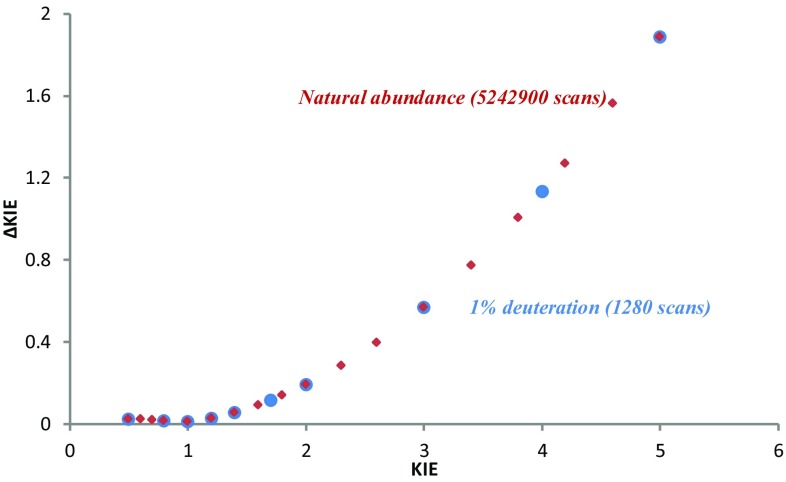
Comparison of error in KIEs with different amount of deuterated substrate and number of scans

When using the methodology developed by Singleton and co-workers with natural abundance of ^2^H roughly 4096 times as much NMR time would be required to obtain comparable errors to those provided using our method (Fig. 5). Note that the number of scans are 5 × 2^n^ as five FIDs are generally collected per sample to minimize issues of spectrometer stability.

## Conclusions

We have developed a model to investigate the effect of introducing small quantities of deuterated substrates on the error in a KIE evaluated using the method developed by Singleton and co-workers. Our simulations predict that only 1–2% of each deuterated substrate is needed to allow a significant reduction in the error ΔKIE compared with natural abundance methods. To obtain comparable accuracies in KIE without the introduction of any deuterated substrates would require extremely lengthy NMR experiments.

## Footnotes

1 For selected examples see: [7], [8], [9].

2 For selected recent examples see: [10], [11], [12], [13]. Example of determination of ^13^C KIEs using isotopically enriched substrates has been reported by Chan J, Lewis AR, Gilbert M, Karwaski M-F, Bennet AJ (2010) Nat Chem Biol 6:405–407.

3 For selected examples see: [14], [15], [16], [17], [18].

4 The NMR experiments were measured using a 1.1 M solution of **1**. The ^2^H{^13^C} spectra were recorded at 77 MHz using inverse gate decoupling, 6.2 delays between calibrated π/2 pulses, 2.7 s acquisition time, and 256 scans collecting 8192 complex points. Each spectrum was obtaining by taking the sum of five subspectra; six spectra were recorded for the sample.

5 For D-2 - Δ*I*_1 − d2_/*I*_1 − d2_ = 0.012 (taken as the standard deviation of the ^2^H-NMR integrals from 6 spectra divided by the average integral value), [**1**]/[**1-d**_**2**_] = 98 so a = 1.2 ×10^−4^ from Eq. 12; For D-3 Δ*I*_1 − d3_/*I*_1 − d3_ = 0.009, [**1**]/[**1-d**_**3**_] = 114, so *a* = 0.75 × 10^–4^. An average value of *a* = 1.0 × 10^–4^ was taken.

6 The kinetic simulations were carried out using the software COPASI. See: [21].
